# Topical 5% Imiquimod Sequential to Surgery for HPV-Related Squamous Cell Carcinoma of the Lip

**DOI:** 10.3390/medicina57060563

**Published:** 2021-06-02

**Authors:** Giovanni Pentangelo, Steven Paul Nisticò, Eugenio Provenzano, Giusy Ylenia Cisale, Luigi Bennardo

**Affiliations:** 1 Unit of Oral Surgery, Polla Hospital, 84035 Polla, Italy; spec.pentangelog@aslsalerno.it; 2 Department of Health Sciences, Magna Graecia University, Viale Europa, SNC, 88100 Catanzaro, Italy; luigibennardo10@gmail.com; 3 Unit of Dermatology, Annunziata Hospital, 87100 Cosenza, Italy; eprovenzano0@gmail.com; 4 Department of Physiology and Pharmacology, Sapienza University of Rome, 00100 Rome, Italy; doc.ylenia@gmail.com

**Keywords:** topical imiquimod, SCC, HPV, head and neck cancer

## Abstract

Background: Squamous cell carcinoma (SCC) is one of the most common neoplasms affecting the oral cavity and the face. Its more differentiated forms may be associated with human papilloma virus (HPV) infection. Case report: In this paper, we report the case of an 86-year-old patient with a well-differentiated SCC of the lower lip associated with HPV treated with surgery with a non-complete histological resolution. Imiquimod 5% cream was applied on the surgical scar once a day for two weeks and then once a week. Two years after SCC removal, no relapse has occurred. Conclusions: Topical imiquimod may be a safe and effective treatment after surgery in SCC of the oral area to reduce the risk of relapses.

## 1. Introduction

Head and neck cancer is a term used to define malignant tumors developing on the lips, mouth, nose, and other head and neck areas [1]. The majority of these tumors are squamous cell carcinomas (SCC) [2]. SCC is a tumor characterized by an abnormal and quick growth of keratinocytes in the epidermis [3]. Although this kind of tumor is usually associated with ultraviolet light exposure and tobacco and alcohol use, this type of cancer may be linked to a previous human papilloma virus (HPV) infection in up to 25% of cases [4]. Although the oropharyngeal region is usually the most frequently associated with HPV infection, SCC affects other regions, such as the lip and the tongue, have been associated with this viral infection in up to 37% [5]. Interestingly, types 16 and 18 HPV are usually related to this type of tumor, as it happens in cervical cancers [6,7]. HPV 16 accounts for 90% of cases. HPV-associated cancers tend to have increased survival and better outcomes [8], although SCC affecting the perioral region tends to have a worse prognosis than other types of SCC [9].

## 2. Case Report

**Figure 1:**
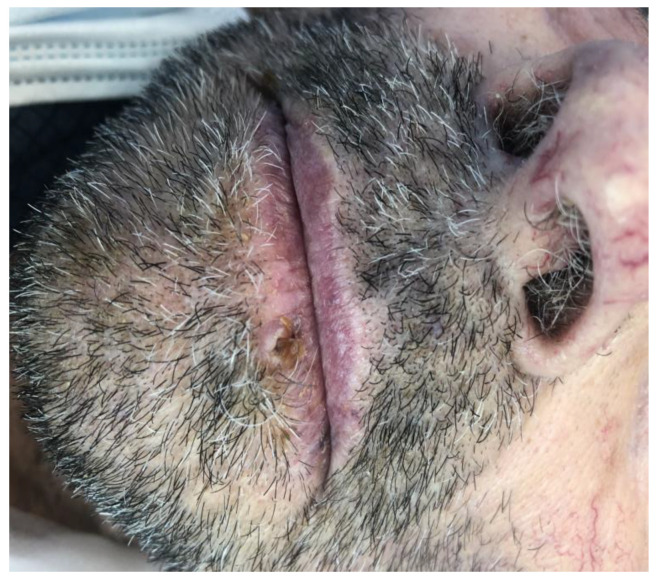
SCC of the lip before surgery.

**Figure 2:**
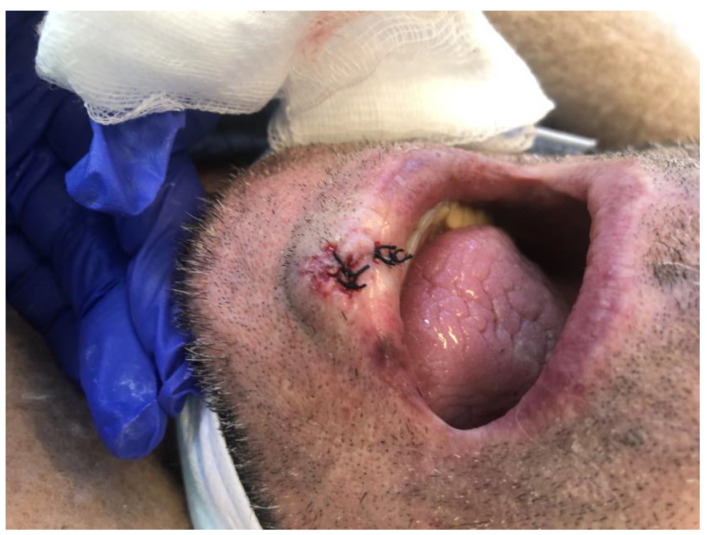
SCC of the lip right after surgery.

An 86-year-old male came to our attention seeking medical treatment for a growing lesion of the lower lip. The patient previously treated the lesion with topical antibiotics and steroids, with no improvement in the condition. The lesion was exophytic and with a warty appearance (Figure 1). Suspecting an SCC, the surgeon performed an excisional biopsy on the lesion. (Figure 2). A total body computerized tomography and an echography of neck and mandibular nodes were performed before surgery, showing no presence of suspect secondarisms.

**Figure 3:**
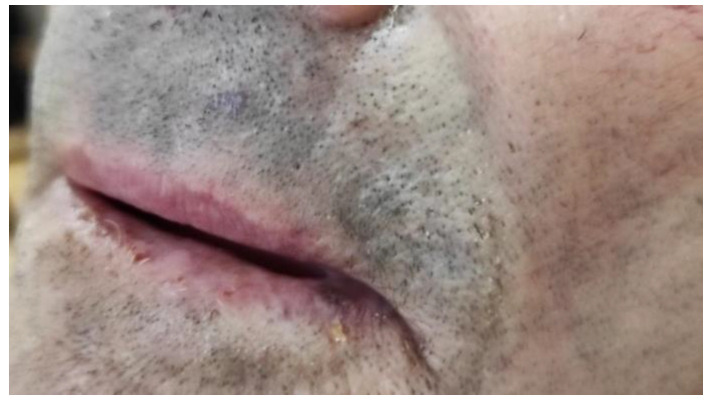
SCC of the lip right after surgery.

**Figure 4:**
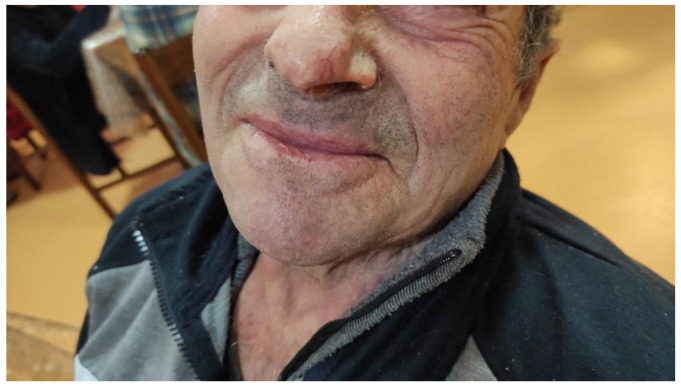
Area affected two years after surgery.

The histopathological examination confirmed the diagnosis of SCC, describing a histologically well-differentiated lesion. HPV typization of the lesion showed positivity for HPV 16 after a conventional polymerase chain reaction assay. The margins of the lesions were not microscopically clear. Considering the patient’s old age and the unwillingness to undergo another surgical procedure, the patient, one month after surgery, was sent to the Dermatological Unit of Magna Graecia University, Catanzaro, for examination (Figure 3). The patient was there treated with topical 5% imiquimod (Aldara, Meda Pharma S.p.A, Milan, Italy) application on the affected area once a day for two weeks, then once a week. The patient performed another total body computerized tomography one and a half years after surgery due to the follow-up of internal malignancy. No signs of SCC-related manifestations were assessed. Two years after the initial surgical procedure, the patient has not developed any sign of systemic or local relapse of the condition (Figure 4).

## 3. Discussion

Imiquimod is a stimulator of the innate immune system that activates toll-like receptors. Cells activated by imiquimod via toll-like receptor-7 secrete cytokines as interferon-α, interleukin-6, and tumor necrosis factor. Imiquimod may activate various immune cells, such as natural killer cells, macrophages, and lymphocytes [10,11]. This drug is topically used to treat actinic keratosis, intraepithelial neoplasias, and some HPV-related lesions such as warts [12].

Due to its high aggressiveness, SCC should always be surgically treated. In cases where surgery is not possible, or in case of comorbidities, other treatments, such as radiotherapy or chemotherapy, are usually proposed [13,14]. Although topical therapies are not usually indicated in the treatment of SCC, various papers have reported the use of topicals in the treatment of SCC [15,16].

Wester et al. reported an 87-year-old female patient affected by an oral SCC twice treated with surgery and subsequent radiotherapy. The lesions reoccurred after treatment and, due to the old age of the patient, a treatment with imiquimod 5% cream was applied with a cotton swab once a day on the affected area for two weeks, and then once a week. Two months after starting therapy with imiquimod, a biopsy in the treated lesions showed no sign of SCC. Four years after the beginning of local applications with imiquimod, no recurrence of the tumor was present [17]. We used the same posology proposed by Wester and colleagues (2017), obtaining similar results, although the affected area was different. This case is, to our knowledge, the first where an SCC of the lip has been treated with topical imiquimod.

Although precancerous lesions of the area, such as actinic cheilitis, have been treated with various topical drugs, such as actixicam, ingenol mebutate, diclofenac, imiquimod, fluorouracil, and photodynamic therapy [18,19,20], the only other topical treatment proposed, also in combination with surgery for lip SCC, was photodynamic therapy. A Chinese group proposed this solution using topical photodynamic therapy after surgery to treat lip SCC in a case series of two patients, with no recurrence reported after two years of follow-up [21].

We believe that combining traditional surgery techniques with a topical treatment such as imiquimod or photodynamic therapy may help reduce the relapse of this condition, as the recurrence of SCC is a common problem.

Limitations to this case include the fact that a biopsy of the region was not performed before or after the treatment with topical imiquimod and that incompletely removed SCC may not relapse independently of the applied topical therapy. However, we believe, due to the location of the condition, that imiquimod contributed to the absence of the condition’s relapse. In addition, testing for oncoproteins E6 and E7 was not performed, so the association of HPV 16 with the development of SCC, although probable, is not certain.

## 4. Conclusions

This case suggests that the combination of surgery and subsequent application of topical 5% imiquimod may reduce the risk of relapses when surgery has not been radical, proposing an alternative treatment in elderly patients or in areas where surgical enlargement may be cosmetically unacceptable, has been refused, or is not possible. Testing SCC for HPV positivity may help select the better treatment available, as HPV-related SCC is usually less invasive [8]. This kind of treatment could also be proposed to selected patients with a superficial disease, no risk of metastasis, and when standard care has been refused or is not possible. We must, however, highlight once more that standard surgical and/or radiotherapy treatment should always be performed if possible and that the use of this topical drug may be proposed as an adjuvant post-surgical treatment. A clinical study with many patients is required to confirm these results, as all the evidence combining two treatment techniques available so far in the literature is limited to small case series/case reports.

## Data Availability

Data are available upon request to the Corresponding Author.

## Abbreviations

SCC: squamous cell carcinoma; HPV: human papilloma virus.
